# The Potential Protective Role of Vitamin D Supplementation on HIV-1 Infection

**DOI:** 10.3389/fimmu.2019.02291

**Published:** 2019-09-25

**Authors:** Natalia Alvarez, Wbeimar Aguilar-Jimenez, Maria T. Rugeles

**Affiliations:** Grupo Inmunovirología, Facultad de Medicina, Universidad de Antioquia (UdeA), Medellín, Colombia

**Keywords:** HIV, vitamin D supplementation, comorbidities, immune modulation, metabolic homeostasis, antibacterial response, parathyroid hormone, bone turnover

## Abstract

HIV infection remains a global and public health issue with the incidence increasing in some countries. Despite the fact that combination antiretroviral therapy (cART) has decreased mortality and increased the life expectancy of HIV-infected individuals, non-AIDS conditions, mainly those associated with a persistent inflammatory state, have emerged as important causes of morbidity, and mortality despite effective antiviral therapy. One of the most common comorbidities in HIV-1 patients is Vitamin D (VitD) insufficiency, as VitD is a hormone that, in addition to its physiological role in mineral metabolism, has pleiotropic effects on immune regulation. Several reports have shown that VitD levels decrease during HIV disease progression and correlate with decreased survival rates, highlighting the importance of VitD supplementation during infection. An extensive review of 29 clinical studies of VitD supplementation in HIV-infected patients showed that regardless of cART, when VitD levels were increased to normal ranges, there was a decrease in inflammation, markers associated with bone turnover, and the risk of secondary hyperparathyroidism while the anti-bacterial response was increased. Additionally, in 3 of 7 studies, VitD supplementation led to an increase in CD4+ T cell count, although its effect on viral load was inconclusive since most patients were on cART. Similarly, previous evidence from our laboratory has shown that VitD can reduce the infection of CD4+ T cells *in vitro*. The effect of VitD supplementation on other HIV-associated conditions, such as cardiovascular diseases, dyslipidemia or hypertension, warrants further exploration. Currently, the available evidence suggests that there is a potential role for VitD supplementation in people living with HIV-1, however, comprehensive studies are required to define an adequate supplementation protocol for these individuals.

## Introduction

Human immunodeficiency virus 1 (HIV-1) infection is one of the most important public health problems worldwide, affecting approximately 38 million people and having caused over 32 million deaths. In 2018, 1.7 million people became infected, whereas 1 million died due to HIV-related causes (1). CD4+ T lymphocytes are the primary target cells of HIV, followed by dendritic cells, monocytes, and macrophages. The acute infection is characterized by the destruction of gut-associated lymphoid tissue (GALT) that harbors a high number of CD4+ effector memory cells. Destruction leads to both anatomical and functional alterations of the gut mucosal barrier, facilitating the passage of commensal microorganisms into the circulation system, which in turn, promotes continuous immune activation. This process leads to immune exhaustion, or the inability to respond to infection leading to the destruction of the immune system and uncontrolled viral replication, resulting in increased tumor rates and opportunistic infections characteristic of acquired immunodeficiency syndrome (AIDS) (2, 3).

HIV-1 infection has also been associated with several metabolic disorders, including vitamin D (VitD) deficiency. Different studies have reported insufficient VitD levels [calcidiol serum levels <30 ng/mL (4–6)] in up to 100% of HIV-1 infected individuals and VitD deficiency [calcidiol serum levels <20 ng/mL (4–6)] in at least 30% of infected individuals (3). Even with combination Antiretroviral Therapy (cART), decreased VitD levels have been associated with comorbidities such as osteoporosis, cardiovascular diseases, type II diabetes mellitus, and infections (i.e., tuberculosis) (3, 7–10) all of which can be explained by looking at the immunomodulatory, anti-inflammatory, and antimicrobial properties of this hormone (11–13).

Alterations in VitD metabolism during HIV-1 infection is associated with an increase in proinflammatory cytokines which block the effect of the parathyroid hormone (PTH) and the hydroxylation of calcidiol in the kidney, preventing the synthesis of active VitD (14–17). Furthermore, certain non-nucleoside reverse transcriptase inhibitors (NNRTIs) and protease inhibitors (PIs) affect the function of hydroxylase enzymes from the Cytochromes P450 (CYP450) complex, inducing a marked decrease in calcitriol production, the active form of VitD (7).

Several trials have explored the beneficial effects of VitD supplementation in VitD deficient HIV-1 infected patients, focusing on the role of immune activation in HIV pathogenesis as well as the modulatory role of VitD. Therefore, this work aims to review the causes and comorbidities related to hypovitaminosis D during infection, with an emphasis on VitD supplementation in HIV-1 infected individuals. Consequently, we conducted a search using different databases such as PubMed, Scopus, Web of Science and Science Direct, with the search terms HIV-1 with vitamin D supplementation, cholecalciferol dose, vitamin D trial, cholecalciferol supplementation, and 25-Hydroxyvitamin supplementation. We excluded case reports, studies with <15 individuals, studies which supplemented with several micronutrients at once or did not report on VitD supplementation, as well as those that were conducted in a non-HIV population. In addition, to control for variability, a supplementation trial was also excluded due to low patient adherence (18).

## Comorbidities During HIV-1 Infection

While the current use of cART has dramatically decreased AIDS-related morbidity and mortality, its long-term use does not lead to viral eradication (19, 20) and is associated with side-effects (21) and viral drug-resistance (22), making long-term management of HIV-1 infection challenging to achieve. Moreover, persons living with HIV-1 often develop complications related to infection and treatment, with increased risk of complications associated with patient lifestyle, aging, and persistent inflammation (characteristic of HIV-1 infection). Complications include diabetes mellitus, chronic kidney disease, cardiovascular disease, and dyslipidemia (23), loss of bone mineral density (24), as well as a higher susceptibility to bacterial infections (such as Tuberculosis, a leading cause of death among people with HIV) (25, 26). However, to date, despite global efforts, interventions to effectively reduce HIV-related inflammation and comorbidities beyond effective and safer cART remain elusive.

The immunological component in HIV-1 pathophysiology suggests that endogenous immunomodulators, such as VitD, may have a beneficial impact on the infection. VitD is a hormone that, in addition to its physiological role on mineral metabolism, has pleiotropic effects on immune regulation. Indeed, one of the most frequent comorbidities during HIV-1 infection is VitD deficiency, highlighting a niche for a potential intervention which could significantly improve patients, health.

## Vitamin D

### Metabolism and Function

Around 90% of VitD is obtained from UVB sunlight, with the remaining amount obtained from diet or nutritional supplementation (6). As was widely explained by a recent review by Jiménez-Sousa et al. (27), the natural process of VitD synthesis occurs in the skin by transforming 7-Dihydrocholesterol into vitamin D3 or cholecalciferol. Subsequently, cholecalciferol is hydroxylated to 25-hydroxycholecalciferol or calcidiol (25OHD) in the liver by the enzyme 25-hydroxylase, which is encoded by the CYP2R1 and CYP27A1 genes. Within the kidney, 1α-hydroxylase, encoded by the CYP27B1 gene, then transforms calcidiol into 1,25-dihydroxycholecalciferol (1,25 (OH) 2D), the physiologically active form of vitamin D (i.e., calcitriol). On the other hand, the enzyme 1,25-dihydroxyvitamin D3 24-hydroxylase, encoded by the CYP24A1 gene, is responsible for initiating calcitriol degradation and regulation.

Calcitriol is the ligand for the VitD receptor (VDR), which is located in the cytosol. Once calcitriol binds the VDR, the complex is translocated into the nucleus where it forms a secondary complex with the retinoid X receptor (RXR). Together, this complex acts as a transcription factor binding specifics sites within the DNA, known as VitD response elements (VDRE), which are located in a significant number of genes, emphasizing their essential role in gene expression regulation (16, 28–30).

VitD function is associated with mineral metabolism as well as bone maintenance. In these processes, VitD directly suppresses PTH release and regulates osteoblast and bone resorption (31). It also improves the absorption of calcium and phosphorus, promoting bone matrix mineralization. Clinical trials have demonstrated an essential role for VitD in preventing osteoporosis, bone breakage, and rickets (32).

Studies have also shown that VDR is expressed on pancreatic β cells as well as on adipocytes indicating a role for calcitriol in insulin secretion and insulin resistance (33). In *in vitro* and *in vivo* cancer therapy experiments, calcitriol has been reported to delay metastasis development by blocking the cell cycle, stimulating DNA repair, and inducing apoptosis (34, 35). VitD also plays a role in cardiovascular diseases, as VDR and CYP27B1 are expressed on myocytes and heart fibroblasts and the inhibition of VDR in mice has been correlated to cardiac hypertrophy (36).

### Effects of Vitamin D on the Immune System

VitD influences both the innate and adaptive immune responses through the expression of its receptor on various immune cells such as monocytes, dendritic cells, and lymphocytes (37–40). VitD modulates the immune system by regulating transcription factors such as NF-AT and NF-kB, and by directly binding VDRE. During the innate response, VitD improves the antimicrobial effects of macrophages and monocytes by promoting transcription of antimicrobial peptides such as defensins (DEF) and cathelicidin (CAMP) (11). Recent research shows enhanced phagocytic and cytolytic activity in VitD-treated macrophages and NK cells, respectively (12, 41).

In addition, during the adaptive response, VitD decreases dendritic cell maturation, reducing the expression of MHC class II and their co-stimulatory molecules (CD40, CD80, and CD86) decreasing their ability for antigen presentation and T cell activation. Therefore, VitD promotes a tolerogenic immune status with a lower inflammatory response, indirectly influencing the polarization of T cells (13). In fact, VitD decreases IL-12 and IFN-gamma production, while increasing IL-10, favoring the development of Th2 and Treg cells over Th1 and Th17 (42, 43). As a result, it has been proposed that VitD promotes tolerance and controls exacerbated immune responses.

### Effects of Vitamin D Deficiency During HIV-1 Infection

Low VitD levels affect individuals of all ages in the general population and is a global issue. Indeed, it has been reported that over 75% of the US population has VitD deficiency (42, 44). Although the VitD deficit is widespread, people living with HIV-1 are more susceptible to hypovitaminosis D, with up to 100% prevalence reported in some HIV-1 infected cohorts across the world; a condition that has been correlated with comorbidities in seropositive individuals (9). In this population, osteopenia and osteoporosis have also been associated with hypovitaminosis D in up to 60 and 20% of infected individuals, respectively (45). Likewise, VitD may also contribute to the increased risk of cardiovascular disease (CVD) reported among HIV-1 infected patients (46). A similar finding has been reported in individuals with diabetes mellitus (10, 47). Lastly, in HIV+ individuals with tuberculosis, VitD deficiency has been associated with a worse clinical outcome (48).

Even though previous studies have associated the levels of VitD with CD4+ T cell recovery in individuals on cART (9, 49), the relationship between VitD deficiency and CD4+ T cell count remains unclear. Moreover, HIV-1 viral load and disease progression have been positively associated with low levels of VitD. Therefore, it is plausible that VitD supplementation may have a beneficial effect on immune recovery, which could decrease comorbidities among HIV-1 infected individuals (50).

## Vitamin D Supplementation in HIV-1 Infected Individuals

Characteristics of the 29 VitD supplementation trials included in this review are listed in Table 1. These studies were carried out in HIV-1 infected individuals, mainly of African-American or Afro-descendants, followed by Caucasians, and had a greater representation of men (60%). The number of individuals recruited for each trial ranged from 17 to 365, all of which were supplemented orally with cholecalciferol (Vitamin D3), except in the study by Falasca et al. in which individuals were also administered supplements via the intramuscular route (59). In approximately half (55%) of the studies, individuals were adherent to a cART regimen, while in the remaining studies, more than 65% of individuals were under a cART regimen and had an undetectable viral load. Prior to supplementation, the average VitD levels were <20 ng/mL, supporting that HIV infected individuals usually suffer severe hypovitaminosis D.

**Table 1:** Vitamin D supplementation studies in HIV-1 infected individuals.

**References**	**Age [mean (range)]**	**(n)**	**The dose used in the study. (*Normalized to daily dose)*. (IU)**	**Control group**	**%Subjects on cART/ virological status**	**Country**	**Ethnic group**	**Efficacy of VitD to restore levels**	**Main results**	**Topic of interest**
Schall et al. (51)	20 (9–25)	58	7,000 daily for 52 weeks	Placebo and before vs. after supplementation	>76/–	USA	84% Black, 16% Hispanic	High	Supplementation was efficient in most participants	Supplementation
Havens et al. (52)	(18–25)	169	50,000 monthly (1,667 daily) for 12 weeks	Placebo and before vs. after supplementation	100	USA	Black 52%, White 22%, Mixed 26%	High	Supplementation was efficient regardless of the cART regimen	Supplementation
Longenecker et al. (53)	47 (39–55)	45	4,000 daily for 12 weeks	Placebo and before vs. after supplementation	100/78% undetectable	USA	78% Black, 15% White, 4% Hispanic, 3% other	Low	Individuals had severe VitD deficiency and did not reach sufficient calcidiol levels. FMD did not change, while PTH levels decreased	Cardiovascular
Muhammad et al. (54)	33 (25–47)	165	4,000 daily for 48 weeks	Placebo and before vs. after supplementation	100 recently	USA	27% Black, 20% Hispanic, 31% White	High	Supplementation did not change the lipid or glucose profile after starting therapy	Metabolic dysregulation
van den Bout-van den Beukel et al. (55)	>18	20	2,000 daily for 14 weeks, then 1,000 daily 48 weeks	Before vs. after supplementation	90	Netherlands	–	High	Insulin sensitivity and PTH levels decreased at week 24 but then returned to baseline levels	Metabolic dysregulation
Chun et al. (56)	<25	102	4,000 or 7,000 daily for 12 weeks	Placebo and before vs. after supplementation	75/50% undetectable	USA	–	High	CAMP expression increased but only 52 weeks after follow-up	Antibacterial response
Lachmann et al. (57)	35	17	200,000 once (6,667 daily) for 4 weeks	Before vs. after supplementation. cART-Naïve and uninfected individuals	65/–	England	18% Black, 63% White, 9% Asian, 9% Indian	High	The levels of CAMP and MIP-β, associated with an anti-HIV-1 effect, increased. Supplementation modestly reduced CD38+ T-cell frequency in HIV-infected patients on cART	Antibacterial response, Immune modulation
Noe et al. (58)	46	243	20,000 weekly (2,857 daily) for 52 weeks	Before vs. after supplementation	100/–	Germany	–	42 −78%	Between 42 and 78% of the individuals reached sufficient VitD levels after supplementation. There was no change in CD4 T cell counts	Immune modulation
Falasca et al. (59)	45 (34–56)	153	300,000 intramuscular every ten months (1,017 daily) or 25,000 oral monthly (892 daily), for 40 weeks	Supplemented vs. unsupplemented individuals	100/–	Italy	White	30–50%	Oral supplementation was more efficient than intramuscular administration; there was no change in CD4 T cell counts	Immune modulation
Fabre-Mersseman et al. (60)	49 (41–54)	53	100,000 every 14 days (7,142 daily) for 48 weeks	Before vs. after supplementation and deficient vs. sufficient individuals	100/–	France	–	High	The activation levels decreased, and the CD4/CD8 T cell ratio increased	Immune modulation
Eckard et al. (61)	20 (15–22)	51	18,000 (642), 60,000 (2,142) or 120,000 (4,285) monthly for 52 weeks	Before vs. after supplementation	100/–	USA	86% Black	71–92%	High doses diminished immune activation and exhaustion	Immune modulation
Stallings et al. (62)	(5–25)	58	7,000 daily per 48 weeks	Placebo and before vs. after supplementation	76/–	USA	85% Black	33–40%	RNA viral load decreased with increasing 25(OH)D, and CD4% and Th naive% were increased; NK% decreased short–term	Immune modulation
Dougherty et al. (63)	19 (8–24)	44	4,000 or 7,000 daily, for 12 weeks	Before vs. after supplementation	82/47% undetectable	USA	Predominantly Black	81%	There was a minimal increase in % CD4+ T cell, a decrease in viral load and the activation profile of CD8+ T cells in individuals receiving cART	Immune modulation
Kakalia et al. (64)	11 (7–15)	53	5,600 or 11,200 weekly (800 or 1600 daily), for 24 weeks	Before vs. after supplementation and Supplemented vs. no supplemented individuals	79/–	Canada	64% Black	67%	67% of the individuals reached sufficient VitD levels after supplementation, but there was no effect on CD4 T cell counts	Immune modulation
Giacomet et al. (65)	19 (14–23)	48	100,000 every 3 months (1,190 daily) for 48 weeks	Placebo and Before vs. after supplementation	85/81% undetectable	Italy	Predominantly white. Black were excluded	80%	There was no effect on CD4+ T cell count. However, the Th17/Tregs ratio decreased	Immune modulation
Coelho et al. (50)	45 (38–50)	97	100,000 weekly (14,285 daily) per 5 weeks; then 16,000 weekly (2,285 daily) for 19 weeks	Before vs. after supplementation and deficient vs. sufficient individuals	100/–	Brazil	53% White	83%	There was an association between CD4+ T cell recovery and VitD increase. Efavirenz use was associated with a higher increase in VitD levels	Immune modulation, Supplementation in cART
Steenhoff et al. (66)	19 (5–60)	60	4,000 or 7,000 daily for 12 weeks	Before vs. after supplementation	100/81% undetectable	Batswana	Black	80%	Only two individuals exhibited hypercalcemia after supplementation. Higher levels of VitD were achieved in individuals treated with efavirenz or nevirapine, compared with individuals treated with PI	Supplementation in cART
Lake et al. (67)	49 (41–55)	122	50,000 twice per week (14,285 daily) for 5 weeks; then 2,000 daily for seven weeks	Before vs. after supplementation	100/–	USA	60% White	81%	Tenofovir use did not affect levels reached after 24 weeks of treatment	Supplementation in cART
Lerma-Chippirraz et al. (68)	47 (41–52)	300	16,000 weekly or every 2 weeks (2,285 or 1,142 daily) for 104 weeks	Before vs. after supplementation	95/–	Spain	84,3% White, 9% Hispanic, Black 3%	82%	In 67% of individuals with secondary hyperparathyroidism, PTH levels decreased	PTH levels
Bañón et al. (69)	44 (22–75)	365	16,000 monthly (533 daily) for 36 weeks	Before vs. after supplementation and Supplemented vs. no supplemented individuals	98/–	Spain	90% White, 1% Black, 9% Hispanic	81%	The risk of secondary hyperparathyroidism decreased	PTH levels
Pepe et al. (70)	50	60	600,000 once (5,357 daily) for 16 weeks	Before vs. after supplementation	100	Italy	White	High	PTH levels decreased, and VitD levels increased regardless of the cART regimen	PTH levels
Havens et al. (71)	(18–25)	169	50,000 monthly (1,667 daily) for 12 weeks	Placebo and before vs. after supplementation	100/–	USA	Black 52%, White 22%, Mixed 26%	High	PTH and bone turnover markers (BAP and CTX) decreased only in individuals supplemented with VitD while on tenofovir	PTH levels and Bone composition
Quirico et al. (72)	46 (35–57)	79	3,200 daily for 96 weeks	Before vs. after supplementation	100/–	Italy	White	100%	Supplementation did not affect the bone mass but decreased PTH levels	PTH levels and Bone turnover
Puthanakit et al. (73)	(12–20)	24	400 daily for 24 weeks	Before vs. after supplementation	100/–	Thailand	Asian	Low	There was an increase in the BMDZ–score	Bone turnover
Overton et al. (74)	33 (25–47)	165	4,000 daily for 48 weeks	Placebo and before vs. after supplementation	100/ recently	USA	33% Black, 37% White, 25% Hispanic	High	Supplementation plus the start of cART attenuated the increase in bone turnover markers	Bone turnover
Piso et al. (75)	43 (34–52)	96	300,000 once (3,500 daily) for 12 weeks	Before vs. after supplementation	76	Switzerland	–	High	Bone replacement markers (BAP, PYR and DPD) decreased	Bone turnover
Etminani-Esfahani et al. (76)	40 (31–49)	98	300,000 once (3,500 daily) for 12 weeks	Before vs. after supplementation	100/–	Iran	–	100%	Osteocalcin increased in Efavirenz-treated individuals indicating improvement of bone formation	Bone turnover
Arpadi et al. (77)	10 (6–16)	56	100,000 every 2 months (1,785 daily) for 48 weeks	Placebo and before vs. after supplementation	–/36% undetectable	USA	64% Black, 36% Hispanic	High	Supplementation with calcium and cholecalciferol did not affect bone mass accumulation, despite a significant increase in serum calcidiol levels	Bone turnover
Rovner et al. (78)	21 (5–25)	54	7,000 daily for 48 weeks	Placebo and before vs. after supplementation	76/–	USA	86% Black	Low	No change in bone composition in infected children and youth	Bone turnover

*IU, International Units; cART, Combination Antiretroviral Therapy; VitD, Vitamin D; PTH, Parathyroid Hormone; FMD, Flow Mediated Brachial Artery Dilation; CAMP, Cathelicidin; HBD, Human Beta Defensins; MIP-1β, Macrophage Inflammatory Protein beta; PI, Protease Inhibitor; BAP, Bone-Specific Alkaline Phosphatase; CTX, Carboxy-terminal Collagen Crosslinks; BMDZ-score, Body Mass index Z-Scores; PYR, Pyridinolines; DPD, Deoxypyridinium*.

The variables that had the most heterogeneity among study populations were geographic origin and age, although most of the studies were carried out in America and Europe with little representation of the African and Asian continents (Table 1). All age groups were represented, but several trials were focused on infected children and youth due to the expectation that the infection would last longer leading to chronic and more profound immune dysfunction. The main objective in most trials was to determine whether VitD supplementation allowed individuals to attain normal VitD levels in serum. In most of the studies (93%), the effect on comorbidities and the association with CD4+ T cell count and viral load was also evaluated.

### Safe and Efficient Doses of Supplementation

Despite the fact that most HIV-1 infected individuals suffer from hypovitaminosis D, no optimal, and safe supplementation dose has yet been established for this population. Generally, a healthy person should consume between 400 and 600 IU (International Units) of VitD daily to maintain sufficiency. However, currently, the Institute of Medicine recommends a standard dose of 600 IU to maintain the requirements of 97.5% of the population, with 4000 IU as the maximum daily dose (51). The North American Endocrine Society recommends three times the standard dose for cART-adhering individuals living with HIV (6). However, nine trials exceeded the maximum limits without adverse effects or associated toxicity (Table 1). Supplementation represents a risk when an individual has calcidiol (25 (OH) D) levels higher than 100 ng/mL or when serum calcium levels exceed 2.70 mmol/L (51). Usually, in these instances, the skeletal system, cell membrane permeability, and nerve impulses are affected, leading to muscle weakness or spasms, constant fatigue, kidney conditions, as well as digestive symptoms such as nausea and vomiting. Nonetheless, all of the supplementation studies reported herein were shown to be safe.

In the studies reported in this review, the supplementation schemes varied regarding the dose and frequency of administration. To make the data more homogeneous for ease of comparison, daily doses were calculated according to the equivalent in weeks or months used in each trial (Table 1). The daily dose ranged from approximately 400 to 14,000 IU, with 4,000 and 7,000 IU as the most common doses. The duration of each trial varied from 4 to 104 weeks. Although most of the doses increased VitD levels, sufficiency was challenging to achieve due to the severe deficiency suffered by the HIV-1 infected population. The use of 7000 IU daily was the most effective dose (51, 56, 60, 63, 66, 78), and restored sufficiency [defined as calcidiol serum levels >30 ng/mL (4)] in 80% of treated individuals with higher levels seen following 12 months of treatment (61). Only 2 of the 215 individuals treated with this regimen had calcidiol levels >90 ng/mL and hypercalcemia (66). Once sufficiency is attained, a maintenance dose guaranteeing stable circulatory VitD levels should be established. Since the follow-up period was short during each of the trials, the long term effects of supplementation are still unclear; therefore, further studies will be required to evaluate the safety of long-term use.

VitD supplementation trials can be confounded by several aspects such as the season in which the study is carried out (78) or skin pigmentation since sunlight can affect vitamin levels. A study performed by Dougherty et al. showed that calcidiol basal levels were lower in individuals in winter than in other seasons (63). Additionally, in a healthy population, individuals with darker skin were reported to require higher doses of cholecalciferol (up to 2000 UI/day) to achieve VitD sufficiency (79). Ancestry may also play a key role in affecting the efficacy of supplementation since a study from Botswana reported that the VitD binding protein (DBP) was lower in plasma of individuals of African descent (1.8 umol/L) (66) compared to those which had an Afro-American background (3.3 umol/L) (80). Other factors, such as drug use as well as malabsorption syndromes and other unknown side effects associated with HIV-1 infection can also affect the results of VitD supplementation. Of note, no ethnic bias was identified during the review of the aforementioned studies as most of the results were obtained in trials which included individuals with varying ethnicities.

### The Effect of Vitamin D Supplementation on CD4+ T Lymphocyte Count and Viral Load

CD4+ T cell counts and viral load are essential indicators for determining the clinical course of HIV-1 infection. However, since the mechanisms by which VitD influences HIV-1 disease progression, morbidity, and mortality are poorly understood, further investigations are required.

Currently, studies have shown that in HIV-1 infected individuals, VitD insufficiency is associated with low CD4+ T cell counts. In Coelho et al. 88% of individuals who had a CD4+ nadir count <50 cells/mm^3^ had VitD insufficiency, while only 6% of participants with a similar nadir had VitD levels within normal range (50). In the same study, 1 ng/mL of calcidiol (25(OH) D) was shown to increase CD4 cell count by 3,3 cells/mm3, suggesting a beneficial role of VitD supplementation on immune recovery. Eckard and Dougherty reported similar results, showing a significant increase in CD4+ count after supplementation (11, 63). Likewise, Stallings et al. reported a reduction in viral load following supplementation (62). However, in other studies, VitD supplementation did not affect CD4+ T cell counts (58, 59, 64, 66).

It is important to note that in supplementation trials in which an increase in the CD4+ T lymphocyte count was observed, participants had remained on a cART regimen; therefore, it has been challenging to establish a causal relationship between VitD supplementation, immune recovery, and virological control. However, in a supplementation study in which 9 of the individuals were not on cART, an increase in CD4+ T cell count and differences in virological control were not seen (63). Although these findings still need to be corroborated, this evidence suggests that VitD may enhance immune recovery and viral control in combination with cART and may serve as an adjuvant to current therapy. Furthermore, none of the supplementation trials reviewed herein reported secondary side effects, supporting the safety of VitD treatment.

### Supplementation Effects on Immune Activation

HIV-1 infected individuals have significantly higher levels of immune activation, even with cART, compared to their uninfected counterparts (81). Additionally, hypovitaminosis D has been associated with an increase in inflammatory markers, both in the general population, and in HIV-1 infected individuals (82, 83), therefore VitD insufficiency may facilitate the persistence of systemic immune activation. Taking into account that immune activation is the main mechanism associated with HIV progression and its associated comorbidities, it is necessary to continue the search for immunomodulators that can return the host to an immune quiescent state. Accordingly, it is interesting to speculate the role that VitD may play in this regard since it has been shown to promote the differentiation of naive T cells into Tregs or Th2 cells, inhibiting the development of Th1, and Th17 cells (13, 84). In fact, Fabre-Mersseman et al. reported that, after supplementing VitD insufficient patients with a dose of 7000 IU daily, immune activation levels, determined by measuring the expression of CD38 and Ki67 in CD8+ T lymphocytes, were reduced and there was an increase in the CD4+/CD8+ T cell ratio (60).

Similarly, in a trial by Eckard et al. looking at different doses of VitD supplementation, CD4+ and CD8+ T cell activation, frequency of inflammatory monocytes (CD14+ CD16+), and expression of PD1+ (an exhaustion marker) in CD4+ T cells decreased significantly in individuals treated with 4000 IU daily for 52 weeks (61). These results are in agreement with those reported by Dougherty et al. which showed a decrease in the percentage of activated cytotoxic T cells (CD8+ CD38+ HLA DR+) following a daily dose of 4000 or 7000 IU of VitD for 12 weeks, (63). These results support VitD supplementation as an adjuvant during routine clinical care of HIV-1 infected patients.

### The Effect of Antiretroviral Therapy on the Response to VitD Supplementation

Although there is evidence suggesting that some antiretrovirals affect VitD metabolism, little is known regarding the effect of VitD supplementation on cART. Non-nucleoside Reverse Transcriptase Inhibitors (NNRTI) have been associated with lower levels of VitD. For example, efavirenz has been suggested to increase VitD catabolism and disrupt 25(OH)D synthesis through the modulation of the cytochrome p450 system, which controls VitD hydroxylation (85–88). However, other trials do not support this hypothesis. Indeed, a study comparing several cART regimens showed that after receiving a daily dose of 4000 or 7000 UI of VitD for 12 weeks, VitD levels were 20 ng/mL higher among individuals on efavirenz compared to all other therapeutic regimens (63). In another study using a similar timeline and supplementation dose schedule, individuals treated with efavirenz reached VitD sufficiency. Of note, variations in baseline VitD levels were not associated with any antiretroviral drug (66). These results suggest that, although efavirenz has been associated with low VitD levels, it is possible to reach sufficient concentrations following supplementation. Moreover, once sufficient levels are reached, efavirenz could have additional benefits related to bone mass, as reported in a supplementation trial in South Africans children (89).

Conversely, zidovudine, a Nucleoside Reverse Transcriptase Inhibitor (NRTI), has been associated with lower levels of vitamin D, while tenofovir has not been associated with deficiency nor insufficiency and neither NRTI has shown significant effects during supplementation trials (9, 59, 90). The Protease inhibitors (PIs) have not yet been correlated with baseline VitD levels or with success or failure to achieve sufficient levels after supplementation (17, 91). No data is currently available for the effect of integrase inhibitors or CCR5 inhibitors on supplementation. In summary, according to previous evidence, the use of cART, even including efavirenz, does not limit the achievable objective of increasing levels of VitD during supplementation trials.

### The Effect of Vitamin D Supplementation on HIV-1 Associated Comorbidities

Hypovitaminosis D has been associated with various comorbidities associated with HIV-1 disease progression resulting in higher mortality rates among infected individuals (92, 93). These individuals have an increased risk of osteomalacia and osteoporosis, notable weight loss, low bone mineral density, and a reduction in muscle mass (78). In contrast, individuals with sufficient VitD levels have a low tendency for skeletal affections; however, the ideal level to minimize risk remains unknown (63).

Although a study in HIV-1 infected individuals with vitamin D deficiency showed a significant reduction in the risk of hypocalcemia after supplementation (58), 3 out of the 5 trials that evaluated bone composition found that, despite an increase in VitD levels following supplementation, bone mass did not change in children and adults (72, 78), even with the addition of calcium (89). In contrast, two studies showed that VitD supplementation decrease biomarkers associated with bone turnover (71, 75) while Etminani-Esfahani et al. reported an increase in Osteocalcin, biomarker associated with bone formation following a single high dose of VitD. Similar results were also noted in other studies (73, 94), where VitD supplementation was found to improve bone composition among HIV-1 infected individuals, albeit, this process might require more time than that seen in previously reported studies.

On the other hand, hypovitaminosis D is related to secondary hyperparathyroidism, a reversible state associated with excessive secretion (>65 pg/mL) of PTH (68), a known cause of decreased bone mineral density (95). Consequently, PTH can be an early indicator of vitamin D deficiency and is an essential criterion for determining if a person requires supplementation (65). Studies evaluating secondary hyperparathyroidism in HIV-1 infected persons are scarce, and as a result, there is little data on the impact of this condition on their clinical status. However, five supplementation trials evaluating PTH levels showed that while VitD levels increased, the levels of PTH decreased during the initial phases of the trials (53, 63, 65, 66, 72, 95).

Finally, although some studies have linked VitD deficiency to hypertension, cardiovascular disease, myocardial infarction, and metabolic syndromes in HIV infected individuals, few studies have evaluated the effect of VitD supplementation on these conditions. In a trial by Chris T Longenecker et al., VitD supplementation in HIV-1 infected patients with hypovitaminosis D did not affect endothelial function, measured by flow-mediated brachial artery dilation (FMD). Furthermore, changes in serum 25(OH)D or FMD were not correlated in the treatment group, although they had not reached sufficient levels of VitD. In Muhammad et al. the authors concluded that VitD supplementation is unlikely to be an effective strategy to attenuate metabolic dysregulation following cART initiation, since lipid and glucose profiles did not improve during treatment (54). These results suggest that VitD supplementation is not enough to avoid the development of these comorbidities, and cannot achieve vitamin sufficiency to improve health conditions (53).

### Vitamin D and Bacterial Infections

VitD plays a key role in the effector activity of innate immune cells in response to microbial infections. During monocytes and macrophages activation, the VDR and the enzyme 1α-hydroxylase (CYP27B1), an activator of vitamin D, are expressed. During the intracrine conversion of the VitD precursor (25(OH)D) to its active form (1,25(OH)2D), it is possible to stimulate the expression of antimicrobial peptides such as cathelicidin (CAMP) and human beta defensins (HBD) (96). Some studies reported that VitD affects autophagy, supporting its anti-microbial properties, for example by promoting *Mycobacterium tuberculosis* clearance and antiviral responses (i.e., inhibiting HIV replication) (97). In a supplementation trial, treatment of HIV-1 infected individuals with VitD promoted CAMP expression, despite requiring longer treatment periods compared to uninfected individuals (56). Similarly, an increase in CAMP and macrophage inflammatory protein beta (MIP-1β) production was also reported in another trial (57). Further studies are needed to evaluate other antimicrobial molecules that can be modulated by vitamin D, such as β-defensin 2 or hepcidin.

## Conclusion

**Figure 1:**
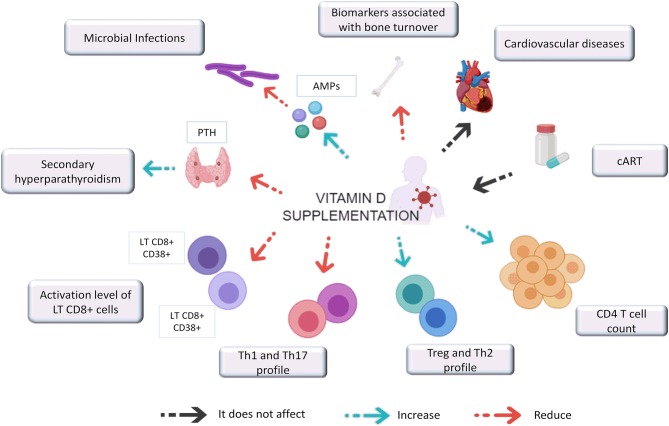
Effect of vitamin D supplementation on clinical and immunological aspects associated with HIV-1 infection. VitD supplementation in HIV-1 infected individuals reduces PTH levels that promotes secondary hyperparathyroidism. It also induces the expression of AMPs (antimicrobial peptides) such as CAMP and HBD and improve bone formation while decrease biomarkers associated with bone turnover. VitD supplementation seems not impact CVD, and the VitD repletion success did not depend on the cART regimen. In addition, supplementation with this hormone seems also to increases CD4 T cell count, promoting their differentiation toward a Th2 and Treg profile while decreasing the Th1 and Th17 profiles and the activation levels of CD8 + T cells.

VitD supplementation in HIV-1 infected individuals leads to an increase in VitD serum levels, regardless of cART, geographical location, and ethnicity of the individual being administered the supplementation. Increased VitD levels may have positive effects on several clinical and immunologic aspects which are summarized in Figure 1. Among them, the most striking results included the potential reduction in the likelihood of secondary hyperparathyroidism and microbial infections such as tuberculosis, as well as an increase in CD4+ T lymphocytes count and a decrease in biomarkers associated with bone turnover and chronic inflammation. However, the effect of VitD supplementation on viral load has not yet been established since the current guidelines for HIV patient management indicate initiation of therapy as soon as individuals are diagnosed, making it impossible to evaluate. Furthermore, the effect of VitD supplementation on the incidence of other comorbidities associated with hypovitaminosis D, such as metabolic syndromes has not yet been carried out.

Overall, evidence suggests that VitD supplementation may be a good adjuvant to cART. However, it is important to emphasize that the effects greatly depend on the dose quantity and duration of which the supplementation is given. In general, the dosages which showed the most success were 4000 and 7000 IU daily for at least 12 weeks. Studies with larger sample sizes are required to confirm the beneficial effects of VitD and to establish optimal supplementation and maintenance doses in the context of HIV-1 infection.

## Author Contributions

NA contributed with the literature search and reading and writing and correcting the manuscript. WA-J contributed with writing and suggestions and corrections. MR contributed to reviewing the manuscript and writing.

### Conflict of Interest

The authors declare that the research was conducted in the absence of any commercial or financial relationships that could be construed as a potential conflict of interest.

## References

[1] UNAIDS *Data 2019*. Jt United Nations Program HIV/AIDS. Geneva (2019). p. 1–468. Available online at: https://www.unaids.org/sites/default/files/media_asset/2019-UNAIDS-data_en.pdf

[2] Alcamí J Coiras M Inmunopatogenia de la infección por el virus de la inmunodeficiencia humana. Enferm Infecc Microbiol Clin. (2011) 29:216–26. 10.1016/j.eimc.2011.01.006 21388715

[3] Barbosa N Costa L Pinto M Vitamin D and HIV Infection : a systematic review. Immunod Disord. (2014) 3:1 10.4172/2324-853X.1000107

[4] Thacher TD Clarke BL. Vitamin D insufficiency. Mayo Clin Proc. (2011) 86:50–60. 10.4016/26528.01 21193656 PMC3012634

[5] Holick MF. Vitamin D Status : measurement, interpretation, and clinical application. Elsevier. (2009) 19:73–8. 10.1016/j.annepidem.2007.12.001 18329892 PMC2665033

[6] Holick MF Binkley NC Bischoff-Ferrari HA Gordon CM Hanley DA Heaney RP. Evaluation, treatment, and prevention of vitamin D deficiency: an endocrine society clinical practice guideline. J Clin Endocrinol Metab. (2011) 96:1911–30. 10.1210/jc.2011-0385 21646368

[7] Conesa-Botella A Florence E Lynen L Colebunders R Menten J Moreno-Reyes R. Decrease of vitamin D concentration in patients with HIV infection on a non nucleoside reverse transcriptase inhibitor-containing regimen. AIDS Res Ther. (2010) 7:40. 10.1186/1742-6405-7-40 21092280 PMC3001414

[8] Sudfeld CR Wang M Aboud S Giovannucci EL Mugusi FM Fawzi WW. Vitamin D and HIV progression among Tanzanian adults initiating antiretroviral therapy. PLoS ONE. (2012) 7:e40036. 10.1371/journal.pone.0040036 22768212 PMC3386915

[9] Lake JE Adams JS. Vitamin D in HIV-infected patients. Curr HIV/AIDS Rep. (2011) 8:133–41. 10.1007/s11904-011-0082-8 21647555 PMC3666828

[10] Pinzone MR Di Rosa M Malaguarnera M Madeddu G Focà E Ceccarelli G. Vitamin D deficiency in HIV infection: an underestimated and undertreated epidemic. Eur Rev Med Pharmacol Sci. (2013) 17:1218–32. Available online at: https://www.europeanreview.org/article/4071 23690192

[11] Prietl B Treiber G Pieber TR Amrein K. Vitamin D and immune function. Nutrients. (2013) 5:2502–21. 10.3390/nu5072502 23857223 PMC3738984

[12] Radovic J Markovic D Velickov A Djordjevic B Stojnev S Vitamin D immunomodulatory effect. Acta Med Med. (2012) 51:58–64. 10.5633/amm.2012.0409s

[13] Baeke F Takiishi T Korf H Gysemans C Mathieu C. Vitamin D: modulator of the immune system. Curr Opin Pharmacol. (2010) 10:482–96. 10.1016/j.coph.2010.04.001 20427238

[14] Beard JA Bearden A Striker R. Vitamin D and the anti-viral state. J Clin Virol. (2011) 50:194–200. 10.1016/j.jcv.2010.12.006 21242105 PMC3308600

[15] Ross AC McComsey GA The role of vitamin D deficiency in the pathogenesis of osteoporosis and in the modulation of the immune system in HIV-infected patients. Clin Rev Bone Miner Metab. (2012) 10:277–87. 10.1007/s12018-012-9131-0

[16] Conrado T Miranda-Filho DDB Bandeira F. Vitamin D deficiency in HIV-infected individuals: one more risk factor for bone loss and cardiovascular disease? Arq Bras Endocrinol Metabol. (2010) 54:118–22. 10.1590/S0004-27302010000200006 20485899

[17] Orkin C Wohl DA Williams A Deckx H. Vitamin D deficiency in HIV: a shadow on long-term management? AIDS Rev. (2014). 16:59–74. Available online at: https://www.aidsreviews.com/resumen.php?id=1258&indice=2014162&u=unp 24759452

[18] Benguella L Arbault A Fillion A Blot M Piroth C Denimal D. Vitamin D supplementation, bone turnover, and inflammation in HIV-infected patients. Med Mal Infect. (2018) 48:449–56. 10.1016/j.medmal.2018.02.011 29661598

[19] Arco A Teira R Bachiller P Pedrol E Domingo P Mariño A Late initiation of HAART among HIV-infected patients in spain is frequent and related to a higher rate of virological failure but not to immigrant status. HIV Clin Trials. (2011) 12:1–8. 10.1310/hct1201-1 21388936

[20] Piconi S Trabattoni D Gori A Parisotto S Magni C Meraviglia P Immune activation, apoptosis and Treg activity are associated with persistently reduced CD4 R T-cell counts during antiretroviral therapy. AIDS. (2010) 24:1991–2000. 10.1097/QAD.0b013e32833c93ce 20651586

[21] Waal D Cohen K Maartens G Systematic review of antiretroviral-associated lipodystrophy : lipoatrophy, but not central fat gain, is an antiretroviral adverse drug reaction. PLoS ONE. (2013) 8:e63623 10.1371/journal.pone.0063623 23723990 PMC3665842

[22] Maggiolo F Airoldi M Kleinloog HD Callegaro A Ravasio V Arici C Effect of adherence to HAART on virologic outcome and on the selection of resistance-conferring mutations in NNRTI- or PI-treated patients. HIV Clin Trials. (2007) 8:282–92. 10.1310/hct0805-282 17956829

[23] Perturbations I HIV and cardiovascular disease : role of immunometabolic perturbations. Physiology. (2018) 33:74–82. 10.1152/physiol.00028.2017 29212894

[24] Mirza FS Luthra P Chirch L. Endocrinological aspects of HIV infection. J Endocrinol Invest. (2018) 41:881–99. 10.1007/s40618-017-0812-x 29313284

[25] Shahcheraghi SH Ayatollahi J Niri MD Fazilati A The most common bacterial infections in HIV-infected patients Addressing the issue of shortage of oral cholera vaccines on the global front. Medical Journal of Dr. D.Y. Patil University. (2016) 9:773–4. 10.4103/0975-2870.194234

[26] Currier JS Havlir D V. CROI 2018 : complications of HIV infection and antiretroviral therapy. Top Antivir Med. (2018) 26:22–9. Available online at: https://www.iasusa.org/wp-content/uploads/2010/04/18-2-57.pdf 29727294 PMC5963934

[27] Jiménez-sousa MÁ Martínez I Medrano LM. Vitamin D in human immunodeficiency virus infection: influence on immunity and disease. Front Immunol. (2018) 9:458. 10.3389/fimmu.2018.00458 29593721 PMC5857570

[28] Christakos S Dhawan P Verstuyf A Verlinden L Carmeliet G. Vitamin D: metabolism, molecular mechanism of action, and pleiotropic effects. Physiol Rev. (2016) 96:365–408. 10.1152/physrev.00014.2015 26681795 PMC4839493

[29] Cooper C Thorne A. Vitamin D supplementation does not increase immunogenicity of seasonal influenza vaccine in HIV-infected adults. HIV Clin Trials. (2011) 12:275–6. 10.1310/hct1205-275 22180525

[30] Christakos S Ajibade D V Dhawan P Fechner AJ Mady LJ. Vitamin D: metabolism. Endocrinol Metab Clin North Am. (2010) 39:243–53. 10.1016/j.ecl.2010.02.002 20511049 PMC2879391

[31] Escaffi FMJ Miranda CM Alonso KR Cuevas M A. Dieta mediterránea y Vitamina d como potenciales factores preventivos del deterioro cognitivo. Rev Médica Clínica Las Condes. (2016) 27:392–400. 10.1016/j.rmclc.2016.06.012

[32] Bikle DD. Vitamin D metabolism, mechanism of action, and clinical applications. Chem Biol. (2014) 21:319–29. 10.1016/j.chembiol.2013.12.016 24529992 PMC3968073

[33] Kayaniyil S Vieth R Retnakaran R Knight JA Qi Y Gerstein HC. Association of vitamin D with insulin resistance and beta-cell dysfunction in subjects at risk for type 2 diabetes. Diabetes Care. (2010) 33:1379–81. 10.2337/dc09-2321 20215450 PMC2875459

[34] Chung M Lee J Terasawa T Lau J Trikalinos TA. Vitamin D with or without calcium supplementation for prevention of cancer and fractures: an updated meta-analysis for the U.S. preventive services task force. Ann Intern Med. (2011) 155:827–38. 10.7326/0003-4819-155-12-201112200-00005 22184690

[35] Manson JE Mayne ST Clinton SK. Vitamin D and prevention of cancer - ready for prime time? N Engl J Med. (2011) 364:1385–7. 10.1056/NEJMp1102022 21428761

[36] Chen S Law CS Grigsby CL Olsen K Hong TT Zhang Y. Cardiomyocyte-specific deletion of the vitamin D receptor gene results in cardiac hypertrophy. Circulation. (2011) 124:1838–47. 10.1161/CIRCULATIONAHA.111.032680 21947295 PMC4160312

[37] Korf H Wenes M Stijlemans B Takiishi T Robert S Miani M. 1,25-Dihydroxyvitamin D3 curtails the inflammatory and T cell stimulatory capacity of macrophages through an IL-10-dependent mechanism. Immunobiology. (2012) 217:1292–300. 10.1016/j.imbio.2012.07.018 22944250

[38] Morán-Auth Y Penna-Martinez M Shoghi F Ramos-Lopez E Badenhoop K. Vitamin D status and gene transcription in immune cells. J Steroid Biochem Mol Biol. (2013) 136:83–5. 10.1016/j.jsbmb.2013.02.005 23416105

[39] Kongsbak M Levring TB Geisler C von Essen MR. The vitamin D receptor and T cell function. Front Immunol. (2013) 4: 148. 10.3389/fimmu.2013.00148 23785369 PMC3684798

[40] Barragan M Good M Kolls JK. Regulation of dendritic cell function by vitamin D. Nutrients. (2015) 7:8127–51. 10.3390/nu7095383 26402698 PMC4586578

[41] Zasloff M. Antimicrobial peptides of multicellular organisms. Nature. (2002) 415:389–95. 10.1038/415389a 11807545

[42] Adams JS Hewison M. Update in vitamin D. J Clin Endocrinol Metab. (2010) 95:471–8. 10.1210/jc.2009-1773 20133466 PMC2840860

[43] Smolders J Thewissen M Peelen E Menheere P Tervaert JWC Damoiseaux J. Vitamin D status is positively correlated with regulatory T cell function in patients with multiple sclerosis. PLoS ONE. (2009) 4:8. 10.1371/journal.pone.0006635 19675671 PMC2721656

[44] Palacios C Gonzalez L. Is vitamin D deficiency a major global public health problem? J Steroid Biochem Mol Biol. (2014) 144:138–45. 10.1016/j.jsbmb.2013.11.003 24239505 PMC4018438

[45] Bander D Parczewski M Osteoporosis and vitamin D deficiency in HIV-infected patients: genetic and classical factors compared to the HIV-associated ones - review. HIV AIDS Rev. (2012) 11:1–4. 10.1016/j.hivar.2011.11.001

[46] Lai S Fishman EK Gerstenblith G Brinker J Tai H Chen S. Vitamin D deficiency is associated with coronary artery calcification in cardiovascularly asymptomatic African Americans with HIV infection. Vasc Health Risk Manag. (2013) 9:493–500. 10.2147/VHRM.S48388 24009422 PMC3758221

[47] Szep Z Guaraldi G Shah SS Lo Re V Ratcliffe SJ Orlando G. Vitamin D deficiency is associated with type 2 diabetes mellitus in HIV infection. AIDS. (2011) 25:525–9. 10.1097/QAD.0b013e328342fdfd 21178753 PMC3366629

[48] Mansueto P Seidita A Vitale G Gangemi S Iaria C Cascio A. Vitamin D deficiency in HIV infection: not only a bone disorder. Biomed Res Int. (2015) 2015:735615. 10.1155/2015/735615 26000302 PMC4426898

[49] Aziz M Livak B Burke-Miller J French AL Glesby MJ Sharma A. Vitamin D insufficiency may impair CD4 recovery among Women's Interagency HIV Study participants with advanced disease on HAART. AIDS. (2013) 27:573–8. 10.1097/QAD.0b013e32835b9ba1 23095316 PMC3902982

[50] Coelho L Cardoso SW Luz PM Hoffman RM Mendonça L Veloso VG. Vitamin D3 supplementation in HIV infection: effectiveness and associations with antiretroviral therapy. Nutr J. (2015) 14:81. 10.1186/s12937-015-0072-6 26283663 PMC4538921

[51] Schall JI Hediger ML Zemel BS Rutstein RM Stallings VA. Comprehensive safety monitoring of 12-month daily 7000-IU vitamin D3 supplementation in human immunodeficiency virus-infected children and young adults. JPEN J Parenter Enteral Nutr. (2016) 40:1057–63. 10.1177/0148607115593790 26160254

[52] Havens PL Mulligan K Hazra R Flynn P Rutledge B Van Loan MD Serum 25-hydroxyvitamin D response to vitamin D 3 HIV-1 infection. J Clin Endocrinol Metab. (2017) 97:4004–13. 10.1210/jc.2012-2600 PMC3485594 22933542

[53] Longenecker CT Hileman CO Carman TL Ross AC Seydafkan S Brown TT Original article Vitamin D supplementation and endothelial function in vitamin D deficient HIV-infected patients : a randomized placebo-controlled trial. Antiviral Therapy. (2012) 17:613–21. 10.3851/IMP1983 22293363 PMC3898848

[54] Muhammad J Chan ES Brown TT Tebas P Mccomsey GA Melbourne K Vitamin D supplementation does not affect metabolic changes seen with ART initiation. Open Forum Infect Dis. (2017) 4:14–17. 10.1093/ofid/ofx210 PMC5726464 29255724

[55] van den Bout-van den Beukel CJP van den Bos M Oyen WJG Hermus ARMM Sweep FCGJ Tack CJJ. The effect of cholecalciferol supplementation on vitamin D levels and insulin sensitivity is dose related in vitamin D-deficient HIV-1-infected patients. HIV Med. (2008) 9:771–9. 10.1111/j.1468-1293.2008.00630.x 18754805

[56] Chun RF Liu NQ Lee T Schall JI Denburg MR Rutstein RM. Vitamin D supplementation and antibacterial immune responses in adolescents and young adults with HIV/AIDS. J Steroid Biochem Mol Biol. (2015) 148:290–7. 10.1016/j.jsbmb.2014.07.013 25092518 PMC4312738

[57] Lachmann R Bevan MA Kim S Patel N Hawrylowicz C Vyakarnam A. A comparative phase 1 clinical trial to identify anti-infective mechanisms of Vitamin D in people with HIV infection. AIDS. (2015) 29:1127–35. 10.1097/QAD.0000000000000666 25870995 PMC4516350

[58] Noe S Heldwein S Pascucchi R Oldenbüttel C Wiese C Von Krosigk A. Cholecalciferol 20 000 IU once weekly in HIV-positive patients with low vitamin D levels: result from a cohort study. J Int Assoc Provid AIDS Care. (2017) 16:315–20. 10.1177/2325957417702487 28393662

[59] Falasca K Ucciferri C Di Nicola M Vignale F Di Biase J Vecchiet J. Different strategies of 25OH vitamin D supplementation in HIV-positive subjects. Int J STD AIDS. (2014) 25:785–92. 10.1177/0956462414520804 24469972

[60] Fabre-Mersseman V Tubiana R Papagno L Bayard C Briceno O Fastenackels S. Vitamin D supplementation is associated with reduced immune activation levels in HIV-1-infected patients on suppressive antiretroviral therapy. AIDS. (2014) 28:2677–82. 10.1097/QAD.0000000000000472 25493593

[61] Eckard AR O 'riordan MA Rosebush JC Lee ST Habib JG Ruff JH Vitamin D supplementation decreases immune activation and exhaustion in HIV-1-infected youth. Antivir Ther. (2017) 24:347 10.3851/IMP3199 PMC6070412 28994661

[62] Stallings VA Schall JI Hediger ML Zemel BS Tuluc F Dougherty KA. High-dose vitamin D3 supplementation in children and young adults with HIV: a randomized, placebo-controlled trial. Pediatr Infect Dis J. (2015) 34:e32–40. 10.1097/INF.0000000000000483 24988118 PMC4281504

[63] Dougherty KA Schall JI Zemel BS Tuluc F Hou X Rutstein RM. Safety and efficacy of high-dose daily vitamin D3 supplementation in children and young adults infected with human immunodeficiency virus. J Pediatric Infect Dis Soc. (2014) 3:294–303. 10.1093/jpids/piu012 26625449 PMC4854371

[64] Kakalia S Sochett EB Stephens D Assor E Read SE Bitnun A. Vitamin D supplementation and CD4 count in children infected with human immunodeficiency virus. J Pediatr. (2011) 159:951–7. 10.1016/j.jpeds.2011.06.010 21820130

[65] Giacomet V Vigano A Manfredini V Cerini C Bedogni G Mora S. Cholecalciferol supplementation in HIV-infected youth with vitamin D insufficiency: effects on vitamin D status and T-cell phenotype: a randomized controlled trial. HIV Clin Trials. (2013) 14:51–60. 10.1310/hct1402-51 23611825

[66] Steenhoff AP Schall JI Samuel J Seme B Marape M Ratshaa B Vitamin D(3)supplementation in Batswana children and adults with HIV: a pilot double blind randomized controlled trial. PLoS ONE. (2015) 10:e0117123 10.1371/journal.pone.0117123 25706751 PMC4338235

[67] Lake JE Hoffman RM Tseng C-H Wilhalme HM Adams JS Currier JS. Success of standard dose vitamin d supplementation in treated human immunodeficiency virus infection. Open Forum Infect Dis. (2015) 2:ofv068. 10.1093/ofid/ofv068 26125033 PMC4462892

[68] Lerma-Chippirraz E Güerri-Fernández R Villar García J González Mena A Guelar Grinberg A Milagros Montero M. Validation protocol of vitamin D supplementation in patients with HIV-Infection. AIDS Res Treat. (2016) 2016:5120831. 10.1155/2016/5120831 27699068 PMC5028798

[69] Bañón S Rosillo M Gómez A Pérez-Elias MJ Moreno S Casado JL. Effect of a monthly dose of calcidiol in improving vitamin D deficiency and secondary hyperparathyroidism in HIV-infected patients. Endocrine. (2015) 49:528–37. 10.1007/s12020-014-0489-2 25432490

[70] Pepe J Mezzaroma I Fantauzzi A Falciano M Salotti A Di Traglia M. An oral high dose of cholecalciferol restores vitamin D status in deficient postmenopausal HIV-1-infected women independently of protease inhibitors therapy: a pilot study. Endocrine. (2016) 53:299–304. 10.1007/s12020-015-0693-8 26254790

[71] Havens PL Stephensen CB Hazra R Flynn PM Wilson CM Rutledge B. Vitamin D3 decreases parathyroid hormone in HIV-infected youth being treated with tenofovir: a randomized, placebo-controlled trial. Clin Infect Dis. (2012) 54:1013–25. 10.1093/cid/cir968 22267714 PMC3297650

[72] Quirico M Valeria R Lorenza M Umberto P Cristina PM Simona O Bone mass preservation with high-dose cholecalciferol and dietary calcium in HIV patients following antiretroviral therapy. Is it possible? Bone mass preservation with high-dose cholecalciferol and dietary calcium in HIV patients following antiretroviral therapy. Is it possible? HIV Clin Trials. (2018) 19:188–96. 10.1080/15284336.2018.1525841 30445888

[73] Puthanakit T Wittawatmongkol O Poomlek V Sudjaritruk T Brukesawan C. Effect of calcium and vitamin D supplementation on bone mineral accrual among HIV-infected Thai adolescents with low bone mineral density. J Virus Eradic. (2018) 4:6–11. Available online at: http://viruseradication.com/journal-details/Effect_of_calcium_and_vitamin_D_supplementation_on_bone_mineral_accrual_among_HIV-infected_Thai_adolescents_with_low_bone_mineral_density/29568546 10.1016/S2055-6640(20)30234-X PMC5851189

[74] Overton ET Chan ES Brown TT Tebas P McComsey GA Melbourne KM. Vitamin D and Calcium attenuate bone loss with antiretroviral therapy initiation: a randomized trial. Ann Intern Med. (2015) 162:815–24. 10.7326/M14-1409 26075752 PMC4608553

[75] Piso RJ Rothen M Rothen JP Stahl M Fux C. Per oral substitution with 300000 IU vitamin D (Cholecalciferol) reduces bone turnover markers in HIV-infected patients. BMC Infect Dis. (2013) 13:577. 10.1186/1471-2334-13-577 24314015 PMC4029316

[76] Etminani-Esfahani M Khalili H Jafari S Abdollahi A Dashti-Khavidaki S. Effects of vitamin D supplementation on the bone specific biomarkers in HIV infected individuals under treatment with efavirenz. BMC Res Notes. (2012) 5:204. 10.1186/1756-0500-5-204 22537736 PMC3527201

[77] Arpadi SM McMahon D Abrams EJ Bamji M Purswani M Engelson ES. Effect of bimonthly supplementation with oral cholecalciferol on serum 25-hydroxyvitamin D concentrations in HIV-infected children and adolescents. Pediatrics. (2009) 123:e121–6. 10.1542/peds.2008-0176 19117833 PMC3110671

[78] Rovner AJ Stallings VA Rutstein R Schall JI Leonard MB Zemel BS. Effect of high-dose cholecalciferol (vitamin D3) on bone and body composition in children and young adults with HIV infection: a randomized, double-blind, placebo-controlled trial. Osteoporos Int. (2017) 28:201–9. 10.1007/s00198-016-3826-x 27837268

[79] IOM I of M (US) Dietary reference intakes for calcium and vitamin d. Pediatrics. (2011) 130:e1424 10.1542/peds.2012-2590

[80] Powe CE Evans MK Wenger J Zonderman AB Berg AH Nalls M. Vitamin D–binding protein and vitamin D status of black americans and white Americans. N Engl J Med. (2013) 369:1991–2000. 10.1056/NEJMoa1306357 24256378 PMC4030388

[81] Hunt PW Cao HL Muzoora C Ssewanyana I Bennett J Emenyonu N. Impact of CD8 + T-cell activation on CD4+ T-cell recovery and mortality in HIV-infected Ugandans initiating antiretroviral therapy. AIDS. (2011) 25:2123–31. 10.1097/QAD.0b013e32834c4ac1 21881481 PMC3480326

[82] Ansemant T Mahy S Piroth C Ornetti P Ewing S Guilland JC. Severe hypovitaminosis D correlates with increased inflammatory markers in HIV infected patients. BMC Infect Dis. (2013) 13:7. 10.1186/1471-2334-13-7 23295013 PMC3545895

[83] Hyppönen E Berry D Cortina-Borja M Power C. 25-Hydroxyvitamin D and pre-clinical alterations in inflammatory and hemostatic markers: a cross sectional analysis in the 1958 british birth cohort. PLoS ONE. (2010) 5:e010801. 10.1371/journal.pone.0010801 20520739 PMC2875406

[84] Sloka S Silva C Wang J Yong VW. Predominance of Th2 polarization by Vitamin D through a STAT6-dependent mechanism. J Neuroinflammation. (2011) 8:56. 10.1186/1742-2094-8-56 21605467 PMC3118349

[85] Villamor E. A potential role for vitamin D on HIV infection? Nutr Rev. (2006) 64:226–33. 10.1301/nr.2006.may.226-233 16770943

[86] Gyllensten K Josephson F Lidman K Sääf M Severe vitamin D deficiency diagnosed after introduction of antiretroviral therapy including efavirenz in a patient living at latitude 59°N [3]. AIDS. (2006) 20:1906–7. 10.1097/01.aids.0000244216.08327.39 16954738

[87] Hariparsad N Nallani SC Sane RS Buckley DJ Buckley AR Desai PB. Induction of CYP3A4 by efavirenz in primary human hepatocytes: comparison with rifampin and phenobarbital. J Clin Pharmacol. (2004) 44:1273–81. 10.1177/0091270004269142 15496645

[88] Childs K Welz T Samarawickrama A Post FA. Effects of vitamin D deficiency and combination antiretroviral therapy on bone in HIV-positive patients. AIDS. (2012) 26:253–62. 10.1097/QAD.0b013e32834f324b 22112601

[89] Arpadi SM McMahon DJ Abrams EJ Bamji M Purswani M Engelson ES. Effect of supplementation with cholecalciferol and calcium on 2-y bone mass accrual in HIV-infected children and adolescents: a randomized clinical trial. Am J Clin Nutr. (2012) 95:678–85. 10.3945/ajcn.111.024786 22258265 PMC3278244

[90] Fox J Peters B Prakash M Arribas J Hill A Moecklinghoff C. Improvement in vitamin D deficiency following antiretroviral regime change: results from the MONET trial. AIDS Res Hum Retroviruses. (2011) 27:29–34. 10.1089/aid.2010.0081 20854196

[91] Cervero M Agud JL Torres R García-Lacalle C Alcázar V Jusdado JJ. Higher vitamin D levels in HIV-infected out-patients on treatment with boosted protease inhibitor monotherapy. HIV Med. (2013) 14:556–62. 10.1111/hiv.12049 23738846

[92] Mehta S Giovannucci E Mugusi FM Spiegelman D Aboud S Hertzmark E. Vitamin D status of HIV-infected women and its association with HIV disease progression, anemia, and mortality. PLoS ONE. (2010) 5:e8770. 10.1371/journal.pone.0008770 20098738 PMC2808247

[93] Viard JP Souberbielle JC Kirk O Reekie J Knysz B Losso M. Vitamin D and clinical disease progression in HIV infection: results from the EuroSIDA study. AIDS. (2011) 25:1305–15. 10.1097/QAD.0b013e328347f6f7 21522006

[94] Bang UC Kolte L Hitz M Schierbeck LL Nielsen SD Benfield T. The effect of cholecalciferol and calcitriol on biochemical bone markers in HIV type 1-infected males: Results of a clinical trial. AIDS Res Hum Retroviruses. (2013) 29:658–64. 10.1089/aid.2012.0263 23199009

[95] Casado JL Bañon S Andrés R Perez-Elías MJ Moreno A Moreno S. Prevalence of causes of secondary osteoporosis and contribution to lower bone mineral density in HIV-infected patients. Osteoporos Int. (2014) 25:1071–9. 10.1007/s00198-013-2506-3 24057480

[96] Sudfeld CR Mugusi F Aboud S Nagu TJ Wang M Fawzi WW. Efficacy of vitamin D3 supplementation in reducing incidence of pulmonary tuberculosis and mortality among HIV-infected Tanzanian adults initiating antiretroviral therapy: study protocol for a randomized controlled trial. Trials. (2017) 18:66. 10.1186/s13063-017-1819-5 28183335 PMC5301352

[97] Campbell GR Spector SA. Vitamin D inhibits human immunodeficiency virus type 1 and Mycobacterium tuberculosis infection in macrophages through the induction of autophagy. PLoS Pathog. (2012) 8:5 10.1371/journal.ppat.1002689 22589721 PMC3349755

